# The Diamond Route to Safer Aesthetic Surgery

**DOI:** 10.1055/s-0045-1814163

**Published:** 2026-01-05

**Authors:** Nandini Singh Tanwar, Raunak Goyal, Rajat Gupta

**Affiliations:** 1 Department of Plastic Reconstructive and Burns Surgery, All India Institute of Medical Sciences, New Delhi, India; 2 Division of Plastic and Reconstructive Surgery, Brigham and Women's Hospital, Boston, Massachusetts, United States; 3 Department of Plastic and Reconstructive Surgery, RG Aesthetics, Excel Hospital, New Delhi, India

## Introduction

From Plato's time to contemporary neurological research, the concept of beauty has consistently sparked intense debate and profound reflection. The definition of beauty remains ambiguous, varying across individuals and history. It is a growing pursuit, amplified by social media, that highlights its influence on self-esteem and quality of life. 1 Nonetheless, this progress is accompanied by the paramount responsibility to ensure patient safety, which remains the fundamental principle of ethical and effective surgical practice.

Every year, September 17th is celebrated as World Patient Safety Day, which was commenced in the year 2019 by the World Health Organization to recognize patient safety as a global health priority. 2 Patient safety emerged due to the growing complexity of health care systems and the increasing number of patient-related complications. The slogan of the 2024 World Safety Day was “Get it right, make it safe,” highlighting the importance of correct and timely diagnosis to ensure patient safety and improve health outcomes. 3

Globally, India ranks seventh in the number of surgical and nonsurgical aesthetic procedures performed. According to the 2024 global surgery survey performed by the International Society of Aesthetic Plastic Surgeons (ISAPS), the total number of aesthetic procedures performed in India was 12,88,840 cases, with 6,77,040 surgical and 6,11,800 nonsurgical procedures. 4 In comparison with the 2023 global ISAPS survey, there was an increase of 2,60,117 procedures. 5 Aesthetic procedures pose distinct challenges due to the diversity of health care infrastructure and the increasing demand for aesthetic procedures. Although technological advancements and enhanced surgical techniques have contributed to improved outcomes, deficiencies in preoperative evaluation, perioperative protocols, or postoperative care may result in preventable complications. To overcome this, the Indian Society of Anaesthesiologists has laid down preoperative guidelines. 6 While perioperative protocols may vary based on individual cases and institutional practices, strict adherence to standardized guidelines is essential.

Quoting the most recent incident on social media, where a 31-year-old singer and influencer travelled with her husband for her cosmetic surgery to Türkiye in June 2025. There was a commercial agreement to offer her surgery at no cost for promoting the clinic. She underwent breast augmentation, liposuction, and rhinoplasty on Sunday, which was 2 days ahead of the scheduled date of surgery. Hours after her surgery, she had bradycardia followed by cardiac arrest, and even with the best efforts by the anesthesiology team, she could not be revived. 7 There are many such incidents reported every other day, but no conclusive statistics are available in India. Avoid exorbitant or aggressive commercialization of aesthetic procedures. Centers offering freebies, free surgery for free promotions, or overtly cheap procedures should raise suspicion. Surgical practices should run on a strong ethical foundation.

**Fig. 1:**
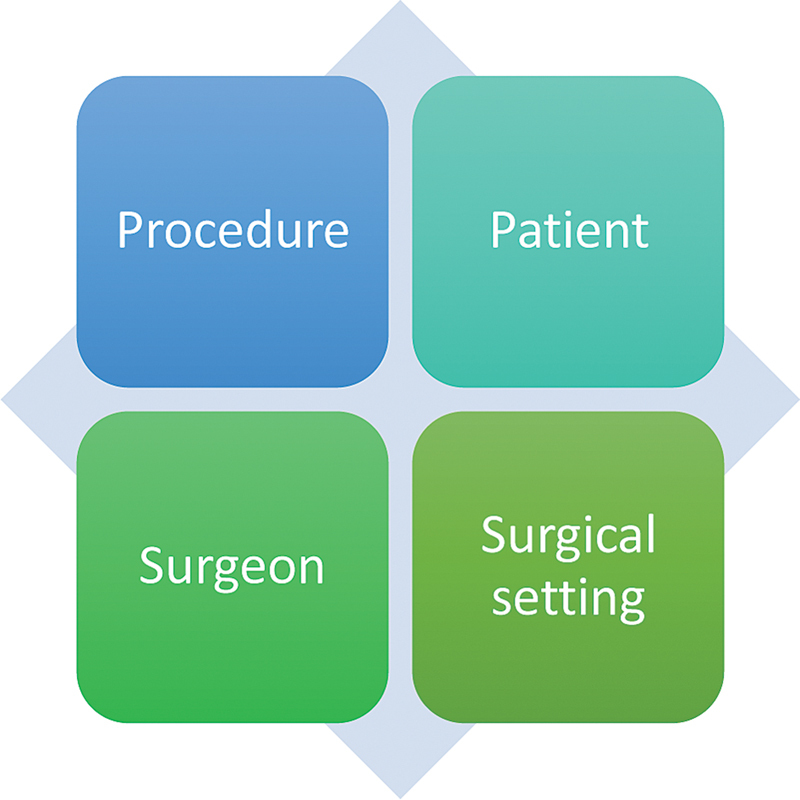
The four diamond factors of patient safety laid down by ISAPS. ISAPS, International Society of Aesthetic Plastic Surgeons.

The surge of complications and incidents, such as surgical site infections, thromboembolic events, anesthetic mishaps, or inadequately managed patient expectations, can compromise the credibility of the specialty. 8 Just as a “diamond” traffic sign warns drivers to anticipate and avoid hazards ahead, the Four Diamond Factors ( Fig. 1 ) in aesthetic surgery established by ISAPS serve as critical safety signals—guiding surgeons and patients to navigate potential risks and ensure a smooth, complication-free journey 9 :

- Procedure: Select procedures appropriate to your goals, supported by thorough research and realistic expectations. Total surgical duration should be within safe limits.
- Patient: A comprehensive medical evaluation is essential to assess risks and overall suitability for aesthetic surgery. Full disclosure of medical history and prior procedures is critical.
- Surgeon: Opt for a board-certified plastic surgeon with proven expertise in your desired procedure and a strong safety track record. Credentials should be verifiable through relevant national medical boards.
- Surgical setting: Every country has a different set of standards. Always ensure the facility is accredited or certified. Hospitals should have recognized certification, and national accrediting bodies should accredit outpatient.

Further disclosure, research, and policy initiatives on patient safety are essential to upholding the standards of our profession and to protect patient welfare.

## References

[1] Sisti A Aryan N Sadeghi P What is beauty? Aesthetic Plast Surg 2021 45 05 2163 2176 33987698 10.1007/s00266-021-02288-2

[2] Binns C Low W Y World patient safety day Asia Pac J Public Health 2020 32(6-7):300 301 32964740 10.1177/1010539520950576

[3] World Health Organization World Patient Safety Day, 17 September 2024: “Improving diagnosis for patient safety.”WHO. Published September 17, 2024. Accessed November 25, 2025 at:https://www.who.int/news-room/events/detail/2024/09/17/default-calendar/world-patient-safety-day-17-september-2024-improving-diagnosis-for-patient-safety

[4] International Society of Aesthetic Plastic Surgery Global statisticsAccessed November 24, 2025 at:https://www.isaps.org/discover/about-isaps/global-statistics/

[5] ISAPS International Survey on Aesthetic/Cosmetic ProceduresAccessed November 26, 2024 at:https://www.isaps.org/media/rxnfqibn/isaps-global-survey_2023.pdf

[6] Preoperative investigations Umesh G Bhaskar S B Harsoor S S Preoperative investigations: practice guidelines from the Indian Society of Anaesthesiologists Indian J Anaesth 2022 66 05 319 343 35782661 10.4103/ija.ija_335_22 PMC9241185

[7] Sukheja B 31-year-old singer-influencer dies after botched cosmetic surgery in TurkeyAccessed June 27, 2025 at:https://www.ndtv.com/world-news/31-year-old-singer-influencer-dies-after-botched-cosmetic-surgery-in-turkey-8717861

[8] Rohrich R J Savetsky I L Avashia Y J Assessing cosmetic surgery safety: the evolving data Plast Reconstr Surg Glob Open 2020 8 05 e2643 33133880 10.1097/GOX.0000000000002643 PMC7572219

[9] ISAPS The patient safety diamondAccessed November 24, 2025 at:https://www.isaps.org/discover/patients-home/safety-considerations

